# Categorical Perception of Control

**DOI:** 10.1523/ENEURO.0258-20.2020

**Published:** 2020-10-14

**Authors:** Wen Wen, Naoto Shimazaki, Ryu Ohata, Atsushi Yamashita, Hajime Asama, Hiroshi Imamizu

**Affiliations:** 1 Department of Precision Engineering, The University of Tokyo, Tokyo 113-8656, Japan; 2 Department of Psychology, The University of Tokyo, Tokyo 113-0033, Japan; 3 Cognitive Mechanisms Laboratories, Advanced Telecommunications Research Institute International, Kyoto 619-0288, Japan; 4 Research into Artifacts, Center for Engineering, The University of Tokyo, Tokyo 113-8656, Japan

**Keywords:** categorical perception, consciousness, motor control, sense of agency, sensorimotor perception, signal detection theory

## Abstract

The self is a distinct entity from the rest of the world, and actions and sensory feedback are our channels of interaction with the external world. This study examined how the sense of control influences people’s perception of sensorimotor input under the framework of categorical perception. Twenty human participants (18 males, two females) took part in both experiments. Experiment 1 showed that the sensitivity (d′) of detecting a 20% change in control from no change was higher when the changes occurred at the control-category boundary than within each category. Experiment 2 showed that the control categories greatly affected early attention allocation, even when the judgment of control was unnecessary to the task. Taken together, these results showed that our perceptual and cognitive systems are highly sensitive to small changes in control that build up to a determinant change in the control category within a relatively narrow boundary zone between categories, compared with a continuous, gradual physical change in control.

## Visual Abstract

**Figure:**
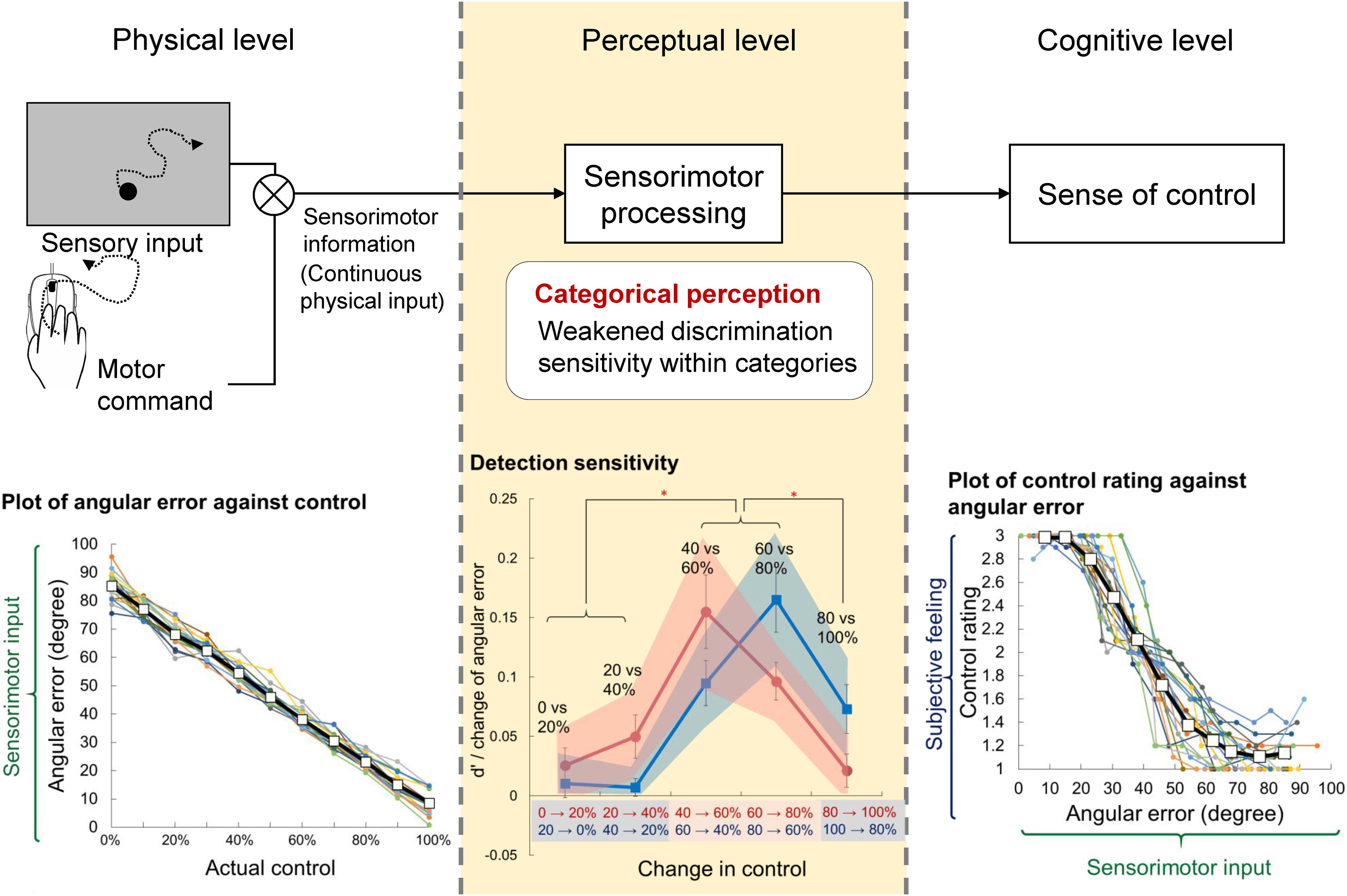

## Significance Statement

Categorical perception is an important cognitive function that connects human low-level perceptual systems with high-level conceptual systems. Categorical perception has been intensively studied with sensory features (e.g., color and faces), but little is known about sensorimotor information, despite its importance for interacting with the external world. This is the first study to show that individuals perceive their control in meaningful categories rather than via linear encoding. The categorical perception of control diminishes sensitivity to differences within control categories, while increasing sensitivity to sensorimotor inputs at the control-category boundaries. The findings broaden our understanding on how human action influences the perception of action consequences, and how humans organize the external world according to the consequences of their actions.

## Introduction

In daily life, people control the objects surrounding them to varying degrees. The subjective feeling of controlling events and changes in the external world, through one’s own actions, is called the sense of control (or, broadly, the sense of agency). The sense of control serves as an important dynamic cue to distinguish the scope of the self, from the rest of the world, as an extension of static self. Things that one controls can be integrated into the scope of the self (Tsakiris et al., 2006; Kao and Goodale, 2009; Salomon et al., 2013). In fact, many psychological studies have shown that the self has a privileged category in our cognitive system (Rogers et al., 1977; Tacikowski and Nowicka, 2010; Salomon et al., 2013). Many types of information, or sensory inputs with certain features, such as name, face, and voice, are associated with the self. These features are usually categorically organized as self versus others, with a clear border between the two categories (Keyes, 2012). However, in the case of control, unlike the passive and static features of the self, dynamic relationships between action and sensory input serve as cues to identify the self. The sensorimotor relationship between motion/action and feedback can be continuous in the physical world. How the cognitive system processes this extension of the self, which is perceived based on the control over the external world, remains largely unknown.

Many existing models for the sense of control emphasize the role of sensorimotor signals. For example, the comparator model suggests that prediction errors, generated from discrepancies between sensory predictions, based on motor commands and actual sensory feedback diminish the sense of control (Blakemore et al., 1998, 1999; Frith et al., 2000a,b; Wolpert and Ghahramani, 2000; Brooks and Cullen, 2013). In addition to the predictive comparison mechanism, a recent study showed that the mechanisms that detect the regular relationship between action and feedback contribute significantly to the sense of control (Wen and Haggard, 2020). However, little is known about the mechanisms linking continuous sensorimotor signals and the cognitive judgment of control.

Humans tend to perceive the world in a meaningful way, using categories of terms that are formed through evolution or learning (Livingston et al., 1998). Many human perceptions are highly categorical, such as color (Bornstein and Korda, 1984), speech (Liberman et al., 1957; Pisoni and Tash, 1974), and facial expression (Etcoff and Magee, 1992; Calder et al., 1996; Young et al., 1997). Early categorization benefits the rapid resolution of ambiguity and efficient decision-making. Once a category is formed, it becomes unnecessary to pay much attention to within-category differences, and decisions based on categories or prototypes can be made smoothly (Etcoff and Magee, 1992). Categorical perception shapes our perception, reducing sensitivity to within-class differences, while increasing the sensitivity to between-class differences (Goldstone and Hendrickson, 2010). Furthermore, previous studies also showed that between-category differences capture visual attention and result in pop-out (Jonides and Gleitman, 1972; Wolfe et al., 1992). This indicates that the influence of categories on perception is rather automatic. In summary, the criterion for categorical perception is how the perceptual sensitivity to a difference/change in the stimuli is shaped by the subjective categories, rather than the cognitive judgment itself (Goldstone, 1994; Goldstone and Hendrickson, 2010).

**Figure 1.**
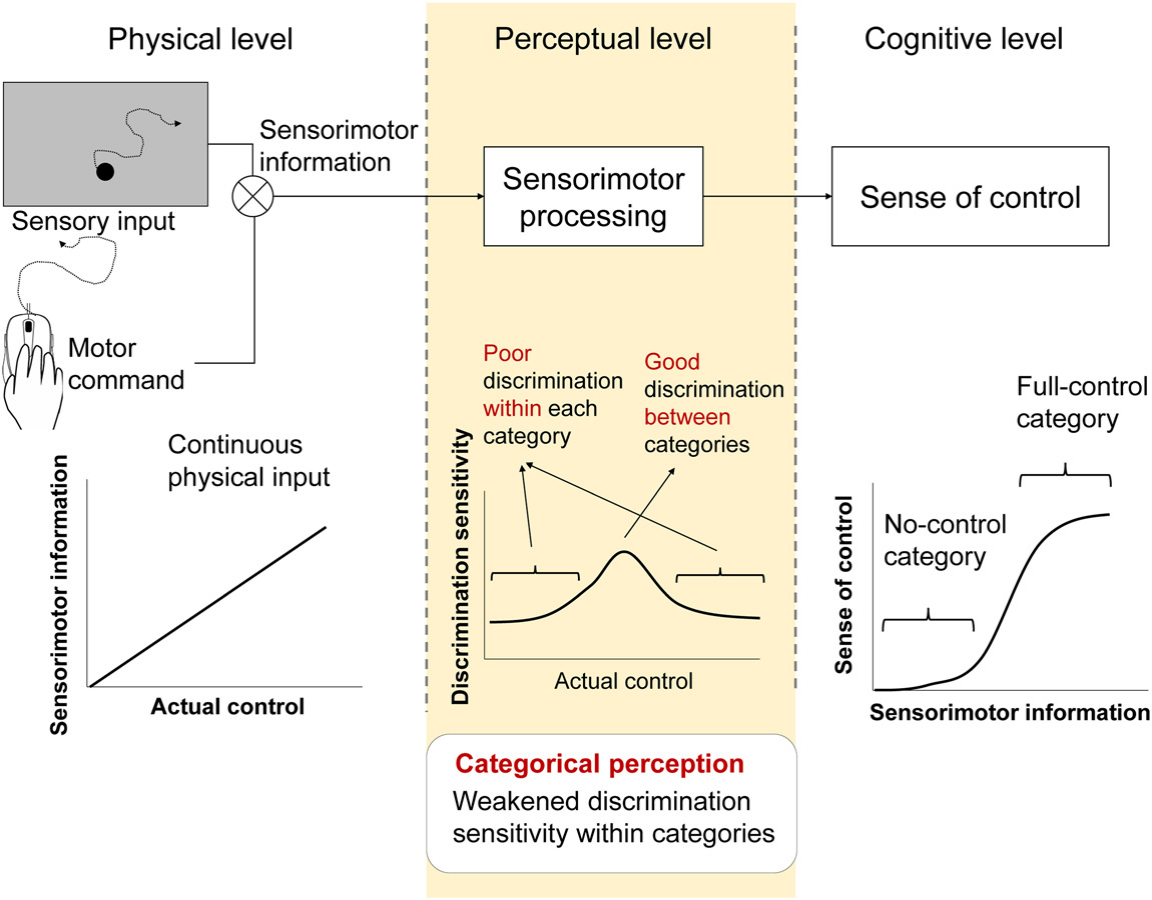
Hypothetical schema of categorical perception of control. Continuous relationship between actual control and sensorimotor information (i.e., on the left) may be organized in a nonlinear fashion at the cognitive level (i.e., on the right), and such categorization at the cognitive level is associated with higher discrimination sensitivity at the boundary between categories, compared with that within each category. In the experimental tasks, participants moved a computer mouse to trigger the motion of a dot on a screen. The controllability of the dot was experimentally manipulated by changing the direction of the dot movement from the mouse movement. The angular error between the mouse and the dot corresponds to the sensorimotor information that determines the sense of control.

Figure 1 shows our hypothetical schema in the present study, that categorical perception of control links continuous sensorimotor signals with cognitive judgment of control. The sensorimotor relationship between action and feedback is continuous in the physical world. On the other hand, many previous studies revealed nonlinear judgment (i.e., categorization at the cognitive level) of the sense of agency (Farrer et al., 2008, 2013; Maeda et al., 2012; Wen et al., 2020). Our hypothesis is that perception of control is categorical. This hypothesis predicts that discrimination sensitivity is high between categories but that the sensitivity is low within a category. We experimentally examined this prediction. Alternatively, if the perception of control is not categorical even when the judgment is nonlinear, then there should be comparable discrimination sensitivities if the distances in sensorimotor input are the same, regardless of whether the differences in sensorimotor input are between or within categories.

This study directly examines the above hypothesis, using a control discrimination task in the first experiment to specifically examine the participants’ ability to detect small changes in control. Furthermore, the second experiment examines whether control categories influence visual attention (Jonides and Gleitman, 1972; Wolfe et al., 1992) while explicit judgment of control is task irrelevant.

## Experiment 1: Detection Sensitivity of a Change in Control

### Materials and Methods

#### Participants

Twenty healthy participants took part in two experiments on different dates (mean age = 21.9, SD = 1.7, 18 males, two females, 18 right-handed participants). Two left-handed participants self-reported daily use of their right hand to manipulate a computer mouse. All participants used their right hands to move the mouse during the tasks. All participants had corrected-to-normal visual acuity. The exclusion criterion for Experiment 1 was a lower detection accuracy than the chance level (<200 correct responses, in 400 trials). One participant met this criterion and was excluded from the results of Experiment 1 (detection accuracy = 39.8%). The experiment was conducted according to the principles of the Helsinki Declaration and was approved by the local ethics committee. All participants provided written informed consent before participation.

#### Motion stimuli

**Figure 2.**
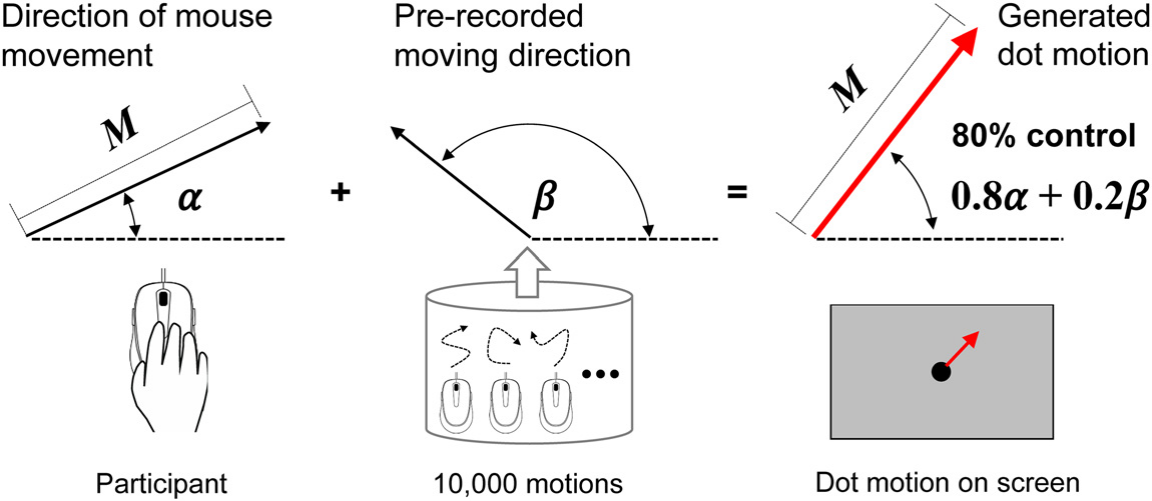
An example of a motion combination algorithm with 80% control. The direction of the visual stimulus (dot) on the screen (red arrow) was generated between the direction of mouse movement (α, in the left panel) and the direction of a prerecorded motion (β, in the middle panel), with an 80/20 ratio at each moment segment (60 Hz). Each direction is represented by the deviation from the right horizontal direction (indicated by dashed lines). The magnitude (M) of the dot movement was equalized to the size of the mouse movement.

A 40-pixel dot was used as the stimulus in the experiment. The dot was presented at the center of the screen (resolution: 1680 × 1050 pixels) at the beginning of each trial, and remained stationary until participants started moving the mouse. The onset, offset, and velocity of the dot motion always corresponded to the mouse’s movement, but the direction of the dot’s motion was a combination of real-time input from the mouse’s motion and 10,000 consecutive prerecorded motions on each refresh frame (60 Hz). Specifically, on each refresh frame, if the mouse’s position changed from the previous frame, the moving angles were calculated according to the mouse’s movement and other prerecorded movements. Depending on the level of control, a moving vector for the dot was generated, with a magnitude corresponding to that of the mouse’s movement, in a direction generated from the angle between the directions of the mouse and prerecorded movements (Fig. 2). Finally, the moving vector was used to update the position of the dot. For example, as shown in Figure 2, for 80% control, the direction of stimulus movement was generated between the angle of the mouse’s movement and the prerecorded motion, at an 80/20 ratio for each moment segment at 60 Hz. Ten thousand motions were prerecorded by the experimenter (Wen et al., 2018), and a randomly selected section was used for each trial/stimulus. This paradigm provided sensorimotor input at the physical level as a linear function of actual level of control, allowing us to examine the potentially discrete or nonlinear perceptual nature of control. The prerecorded other’s movements were recorded mouse movements of the experimenter at each frame (60 Hz) from a pilot recording session, in which the experimenter had full control over the dot (i.e., moving the dot as moving a mouse cursor). Such prerecording ensures smooth and continuous motion of the stimulus and creates joint control between the participants and someone else. In addition, random motion was not suitable for the present paradigm, because it is a lack of continuance and causes vibration-like motion of the stimulus when combined with participants’ mouse movement. In addition, the raw input from the mouse movements was adjusted to 40%, to prevent the stimulus from excessive motion. Finally, when the dot reached the border of the screen, it was blocked by the border (i.e., the dot could not move beyond the border). An algorithm was used to re-select a new section of prerecorded motion that moved the dot toward the inner part of the screen when the dot reached the border area. The algorithm was designed to reduce the possibility of the dot sticking to the border, especially when participants had little control over it. A demo task and source code of this paradigm can be found at the first author’s website (http://www.robot.t.u-tokyo.ac.jp/~wen/code/).

#### Control discrimination task

**Figure 3.**
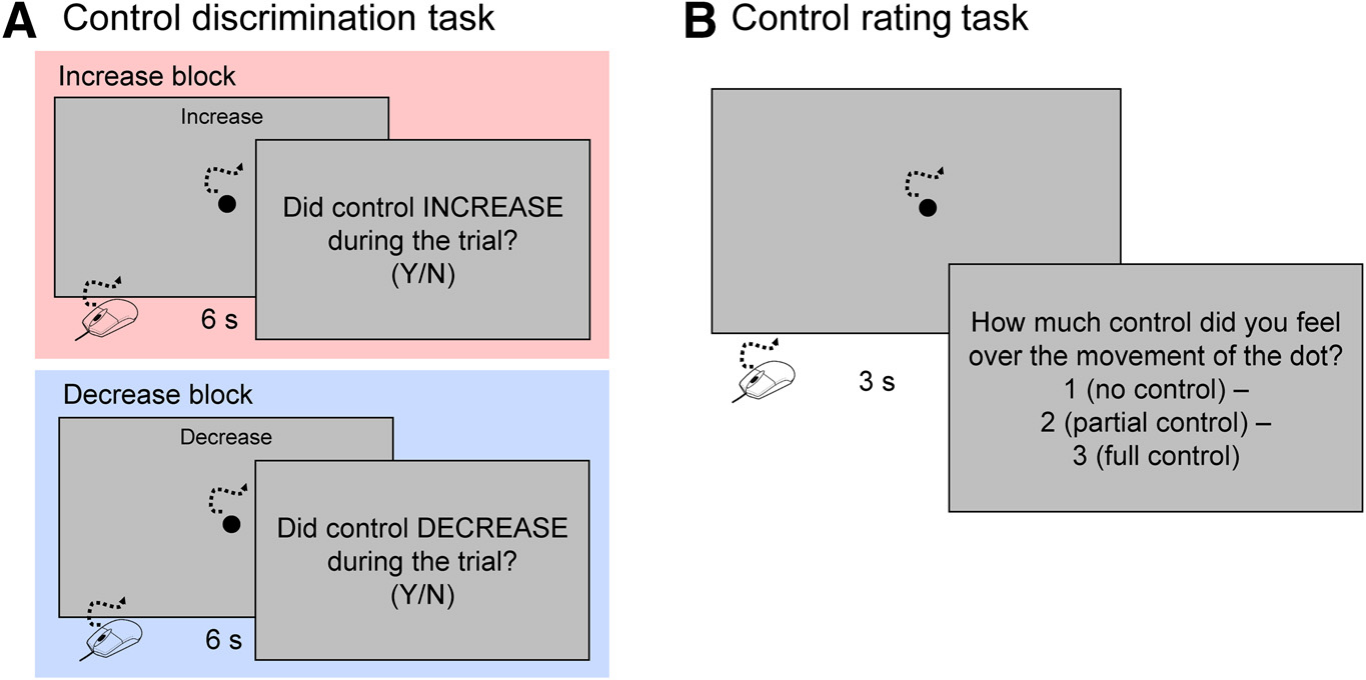
Experimental tasks. ***A***, Control discrimination task. Participants moved a mouse to trigger a 6-s motion of a dot on the screen. At the end of each trial, participants made a binary judgment on whether they felt that their control over the direction of dot’s movement had changed (increased or decreased) during the trial. ***B***, Control-rating task. Participants rated their control over the dot out of three: 1 = no control, 2 = partial control, 3 = full control.

In the control discrimination task of Experiment 1, participants used a computer mouse to move a dot on the screen for 6 s in each trial and made a binary judgment on whether their control over the direction the dot moved had changed (Fig. 3*A*). Participants were instructed to continuously and freely move the mouse to explore their control over the dot on the screen. No specific instructions for movement were given. In half of the trials, the level of control over the dot remained constant, while in the other half, the level of control increased or decreased by 20% (e.g., from 20% to 40% control) halfway through the trial (i.e., 3 s from the onset of the first mouse movement). There were two increase blocks and two decrease blocks in the control discrimination task. The two types of blocks were performed in ABAB sequence, and the sequence of increase/decrease was offset between participants. At the start of each block, a message appeared on the screen, prompting participants if an increase or a decrease in control should be detected. The screen background was gray, and all stimuli and messages were displayed in black. At the start of each trial, the number of current and total trials in the block was displayed on the screen for 1.5 s and then replaced with the stimulus dot. According to the block type, as participants moved the dot, an “Increase” or “Decrease” message was displayed in dark gray at the top of the screen. Six seconds after the onset of the first mouse movement, the dot disappeared and the participants pressed one of two response keys (yes or no), in response to whether they felt their control of the dot change during the trial. The initial control for the increase blocks was 0%, 20%, 40%, 60%, or 80%, and the initial control for the decrease blocks was 20%, 40%, 60%, 80%, or 100%. The magnitude of change was always 20%. The starting points of the prerecorded movements were randomly selected for each trial, and were used to ensure the smooth movement of the dot. Each block consisted of 100 trials (10 repeats of each initial control condition), and the level of control changed in 50 trials, while remaining constant at the initial control level in the other 50 trials. The trial sequence was randomly assigned for each block. A total of 400 trials were conducted in the control discrimination task. The participants performed one increase and one decrease practice block before the actual task. Each practice block consisted of four trials. For the increase practice block, control increased from, or remained constant at, 0% or 60%. In the decrease practice block, control decreased from, or remained constant at, 60% or 100%. The sequence of practice blocks between participants was also offset.

#### Control rating task

The control rating task followed the control discrimination task. In each control rating task trial, participants rated their control of the dot’s direction after moving the dot for 3 s (Fig. 3*B*). At the beginning of each trial, a dot was displayed on the screen, and participants continuously moved a mouse to trigger the movement of the dot. The level of control of the dot was between zero and 100%, in a 10% step range. Three seconds after the first mouse movement, this dot was replaced by a rating indication on the screen. Participants rated their control of the dot, from three options: 1 = no control, 2 = partial control, 3 = full control. Each control condition was repeated 10 times, resulting in 110 trials in total. The trial sequence was randomized for each participant. Participants performed six practice trials before the actual task. For practice, the level of control was 0%, 60%, or 100%, repeated twice each time. The practice trial sequence was randomized.

#### Procedure

Participants performed the experiments individually in a quiet room. They were seated ∼60 cm away from a 22-inch LED monitor (DELL P2217). The response keys on the keyboard were labeled, and the keys that were not required for tasks were removed to prevent accidental responses. Participants first performed the control discrimination task, followed by the control rating task. The first task took approximately 1 h, including instruction and practice, and the second task took ∼8 min.

#### Data analysis

First, we used control ratings to estimate the subjective boundary between no-control and full-control. To do so, the points of subjective equality (PSEs), when participants gave 50% of the trials no-control or full-control ratings, were calculated using logistic regressions with the generalized linear model function of MATLAB, Statistics and Machine Learning Toolbox (R2017a, The MathWorks). Next, we calculated d′ (d-prime) of signal detection theory (Green and Swets, 1966) from the responses, and used a 5 × 2 (comparisons × direction of change) repeated-measures ANOVA to examine whether d′ was indeed higher when the change occurred at the subjective boundary compared with when it occurred within each subjective category. In case of significant main effect or significant interaction, Bonferroni correction of *p* value was used for *post hoc* comparisons, the original *p* values were multiplied by the number of comparisons, and the significance level was 0.05. At last, d′ in each condition was also compared with zero using one-tailed one-sample *t* tests. The Holm–Bonferroni method was used for ten one-sample *t* tests.

### Results

#### Subjective control categories

**Figure 4.**
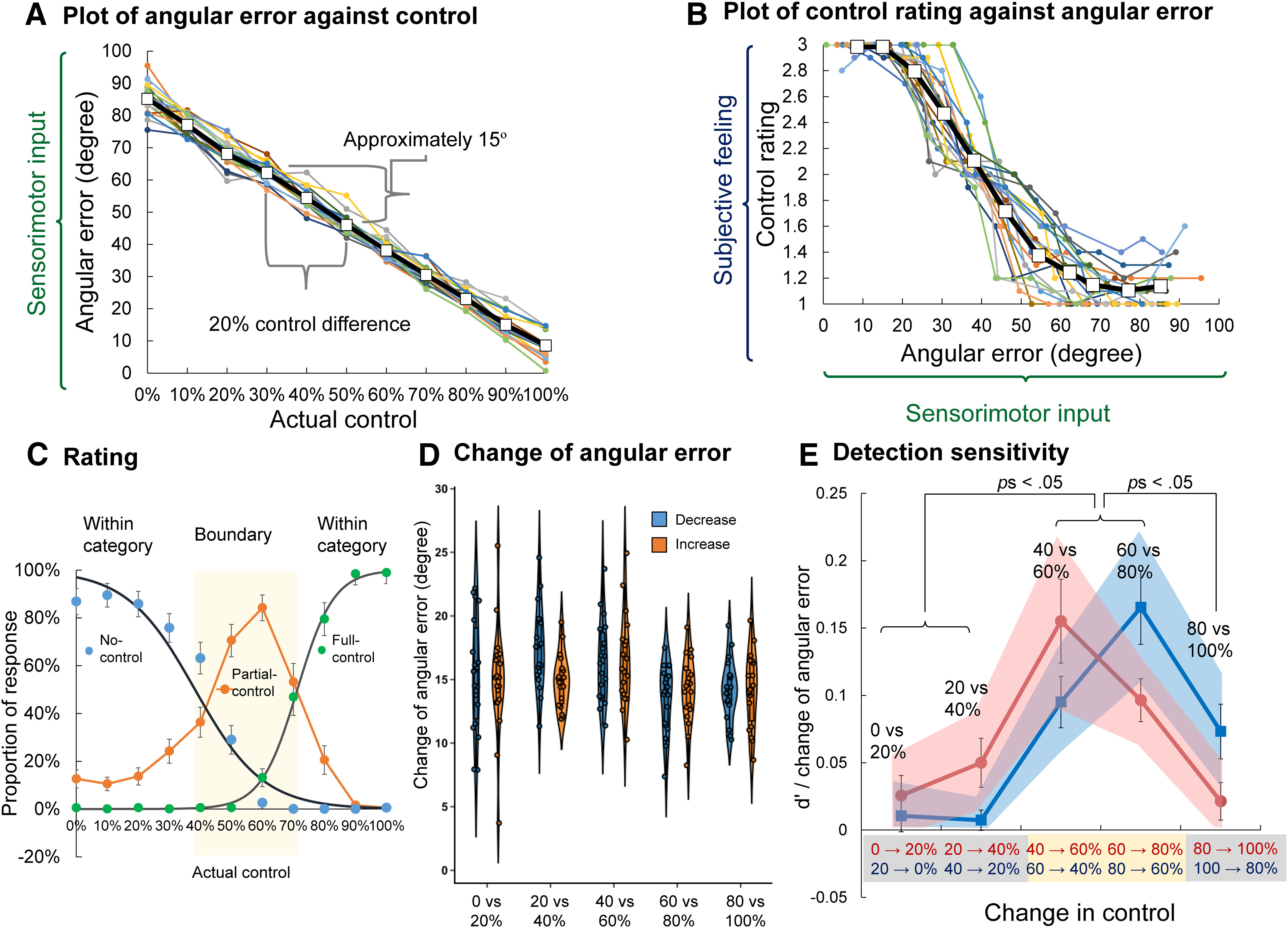
Results of Experiment 1. ***A***, Individual and average plots of angular error against actual control. Colors represent individual results. Open squares and black lines represent averages. These plots indicate that angular error, as a sensorimotor input, is a linear function of the actual level of control. In addition, every 20% control difference corresponds to an angular error difference of ∼15°. ***B***, Individual and average plots of control rating against angular error. Colors represent individual results. Open squares and black lines represent averages. These plots show that the subjective judgment of control is a logistic-like function of continuous sensorimotor input. ***C***, Proportion of each rating response (no control, partial control, and full control). Black curves represent the logistic fitting results for the no-control and full-control responses. The yellow area indicates the range between the no-control and full-control PSEs. The left and right sides of the yellow rectangle indicate the no-control and full-control PSEs (50% threshold), respectively. Error bars represent standard errors. ***D***, Change of angular error under each change condition (i.e., increase or decrease conditions). Under all conditions, the magnitude of control change was 20%. However, the actual change of angular error differed slightly between conditions. Because the physical change of control did not, as expected, remain constant between conditions, d′/ΔE was used (with ΔE representing the change in angular error), instead of raw d′, to exclude the influence of unbalanced physical changes between conditions in all statistical analyses. ***E***, Sensitivity (d′/ΔE) calculated from the response of the control discrimination task. The squares and bold lines indicate averaged d′/ΔE under each condition. Shadows represent 95% confidence intervals. Error bars represent standard errors. Red asterisks represent significant *post hoc* pairwise differences.

First, Figure 4*A* shows the averaged angular error in each actual control condition in the control rating task. The results showed that angular error, as a sensorimotor input, is a linear function of the actual level of control. On the other hand, the subjective rating of control is a logistic-like function of the continuous sensorimotor input (Fig. 4*B*).

Next, the subjective boundary between the no-control and full-control ratings was estimated (Fig. 4*C*). The main interest of the present study was whether perceptual sensitivity (i.e., d′) is higher when a change in control occurs at the boundary of control categories than when it occurs within a control category. The average PSE of no control was 38.0% (SD = 10.9%) and the average PSE of full control was 71.2% (SD = 6.6%; Fig. 4*C*, left and right vertical lines in yellow shaded area). The yellow area is regarded as the boundary between the no-control and full-control categories.

#### Detection sensitivity of a change in control

Next, we investigated the sensitivity to changes in control; d′ of signal detection theory (Green and Swets, 1966) was calculated from the responses as a detection sensitivity index of a 20% change in control from no change. The change in control was kept constant at 20% under all conditions. However, the actual change of sensorimotor input at the physical level differed slightly between conditions (Fig. 4*D*). A 5 × 2 (comparisons × direction of change) repeated-measures ANOVA on the change of angular error revealed a significant main effect of comparison (*F*_(4,72)_ = 4.34, *p *=* *0.003, partial η^2^ = 0.19), and a significant interaction between the comparison and direction of change (*F*_(4,72)_ = 3.54, *p *=* *0.011, partial η^2^ = 0.16). The main effect of direction change was nonsignificant (*F*_(1,18)_ = 0.50, *p *=* *0.491, partial η^2^ = 0.03; M_decrease_ = 15.3°, SD_decrease_ = 1.44°, M_increase_ = 14.94°, SD_increase_ = 0.85°).

To control for the influence of unbalanced sensorimotor inputs, d′ was divided by the actual change in angular error (i.e., d′/ΔE, where ΔE represents the change in angular error; Fig. 4*E*), and this value was used for all statistical analyses. The results of d′/ΔE in Figure 4*E* show peaks of detection sensitivity at the boundaries of the control categories for both a 20% increase and a 20% decrease in control, supporting the prediction of categorical perception of control. A 5 (comparison: 0% vs 20%, 20% vs 40%, 40% vs 60%, 60% vs 80%, or 80% vs 100%) × 2 (change direction: decrease or increase) repeated-measures ANOVA on d′/ΔE revealed a significant main effect of comparison (*F*_(4,72)_ = 20.08, *p* < 0.001, partial η^2^ = 0.53) and a significant interaction between change direction and comparison (*F*_(4,72)_ = 6.45, *p *< 0.001, partial η^2^ = 0.26). The main effect of change direction was nonsignificant (*F*_(1,18)_ = 0.002, *p *=* *0.963, partial η^2^ <0.01). The main effect of comparison showed that the subjective sensitivity to change showed significant differences, depending on whether the change occurred within, or between, control categories. Because the subjective boundaries of the control categories were between 38.0% (SD = 10.9%) and 71.2% (SD = 6.6%), changes between 40% and 60%, and between 60% and 80%, are considered to be at the boundary. *Post hoc* comparisons confirmed that the d′/ΔE in these conditions was significantly larger than for the other conditions. For the decrease condition, the d′/ΔE of 80% to 60% change was significantly larger than 20% to 0%, 40% to 20%, and 100% to 80% changes (*p* < 0.001, *p *=* *0.002, *p *=* *0.042, respectively), but did not significantly differ from the detection of a decrease from 60% to 40% (*p *=* *0.131). Under the increase condition, the d′/ΔE of 40% to 60% change was significantly larger than the 0% to 20%, 20% to 40% and 80% to 100% changes (*p *= 0.004, *p *=* *0.038, and *p *=* *0.009, respectively). However, it did not significantly differ from the detection of an increase from 60% to 80% (*p* > 0.999). In summary, the results of d′/ΔE revealed that detection sensitivity to small changes in control was higher at the boundaries of the control categories than within the same control category, clearly supporting the hypothesis of categorical perception of control.

Furthermore, the significant interaction between comparison and direction of change of the ANOVA revealed an effect of change direction in control on detection sensitivity. The d′/ΔE of detecting a decrease from 100% to 80% control was significantly above zero (*t*_(18)_ = 3.60, *p *=* *0.002, Cohen’s *d* = 0.83). However, the d′/ΔE to detect an increase from 80% to 100% control was not significantly different from zero (*t*_(18)_ = 1.53, *p *=* *0.144, Cohen’s *d* = 0.35). This indicates that, when participants initially had higher levels of control (in the category of full control), they could detect changes toward the boundaries of control but were insensitive to changes toward the current control category extremes. This phenomenon was also observed when people initially had low levels of control (in the category of no control). Specifically, the d′/ΔE of detecting an increase from 20% to 40%, was significantly above zero (*t*_(18)_ = 2.74, *p *=* *0.013, Cohen’s *d* = 0.63). However, the d′/ΔE of detecting a decrease from 40% to 20%, did not significantly differ from zero (*t*_(18)_ = 0.97, *p *=* *0.347, Cohen’s *d* = 0.22). This phenomenon indicates that the establishment of a subjective control category selectively biases the detection sensitivity, according to the direction of change.

In summary, the results of Experiment 1 support the hypothesis of categorical perception of control as shown in Figure 1. Experiment 2 further examined whether the influence of control categories held when the cognitive judgment of control was task irrelevant.

## Experiment 2

### Materials and Methods

#### Participants

The participants were the same as those in Experiment 1. Experiment 2 took place at least one week after Experiment 1. The exclusion criterion for Experiment 2 was <75% of the valid trials (i.e., trials with correct target identification and RTs within ±3 SD from individual average). No participant met this criterion.

#### Stimuli and task

**Figure 5.**
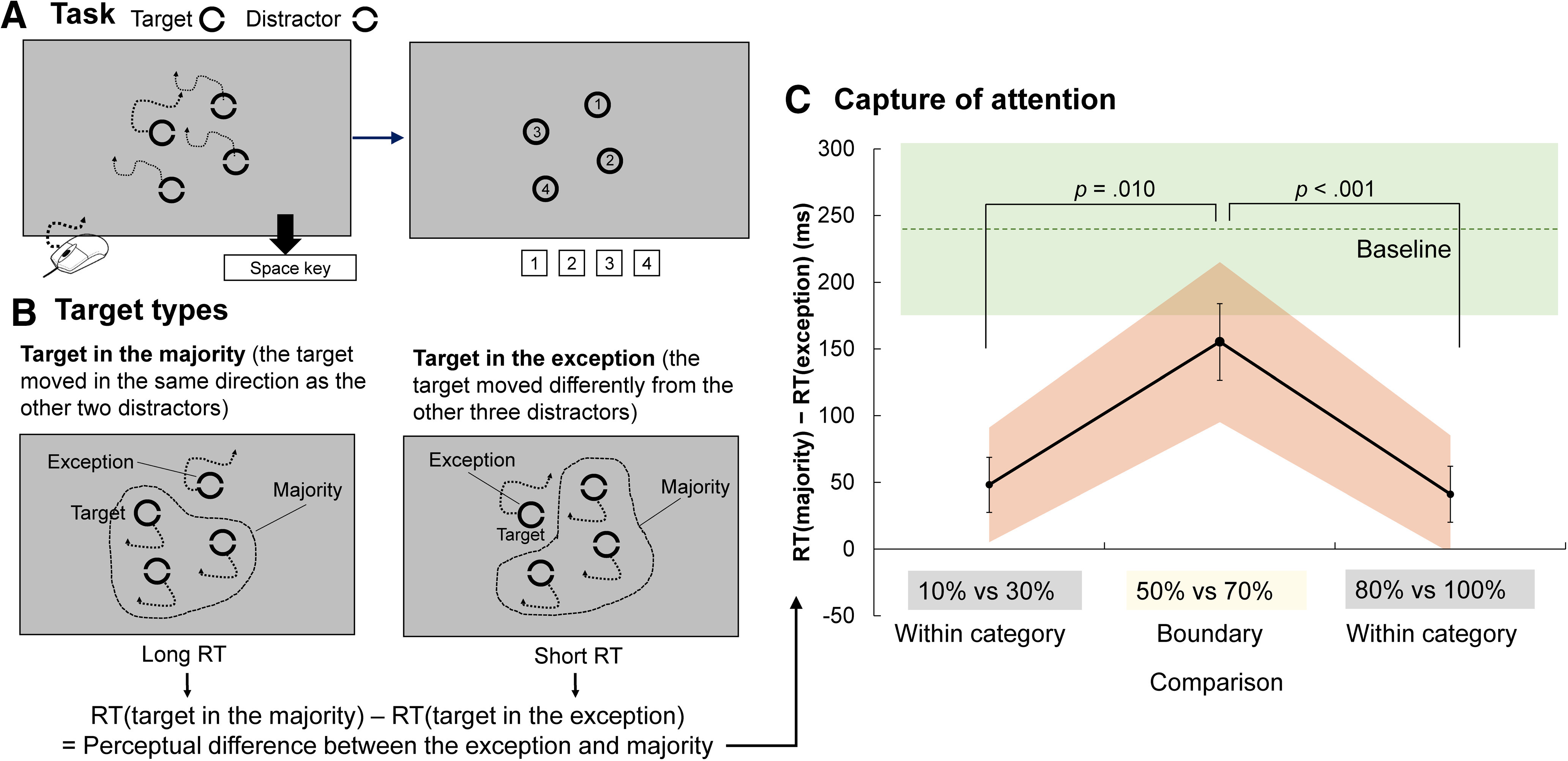
Visual search task of Experiment 2 and the effect of target type on RT. ***A***, Visual search task. Participants moved a mouse to trigger the movement of four circles to search for a circle containing one gap to the right (i.e., the visual target) as quickly as possible, and identified the visual target by pressing a number key. ***B***, Target type (majority or exception) in the visual task. The difference in RT for target detection between the two target types reflects the perceptual difference between the majority and exception stimuli. ***C***, Capture of attention measured by difference in RT between exception and majority target conditions. Shadows represent 95% confidence intervals. Error bars represent standard errors. Red asterisks represent significant *post hoc* pairwise differences. Dashed lines and green shading represent the baseline condition and its 95% confidence interval, respectively.

In the visual search task of Experiment 2, participants were asked to locate a circle with one gap on the right (i.e., the visual target), from a total of four circles (Fig. 5*A*). The other three circles had gaps on the left and right. The circles were 60 pixels, and the widths of the circle borders and gaps were both 10 pixels. In each trial, the initial positions of the circles were randomly generated to meet the requirement that the distance from the center of the screen to each circle was <350 pixels, and the minimum distance between each circle was at least 150 pixels. There were no gaps in the circles, unless they were moved. Participants were asked to move the circles by moving the computer mouse, while searching for the visual target. The onset, offset, and velocity of all circles corresponded to the mouse’s movement, and the motion of the mouse had specific levels of control over the directions of the circles. Participants were asked to press the space bar as soon as they found the visual target, which stopped the moving circles so that the gaps disappeared. Numbers from one to four were displayed in the center of each circle. Participants then pressed one of the four number keys (1–4) to identify the visual target. If there was no response 5 s after the start of the first mouse movement, the trial ended and, in this case, the visual target was skipped.

When participants moved the mouse, three out of four circles always moved in a coherent direction, while one moved slightly differently to the others. The group in coherent motion was called the majority, and the stimulus that moved differently was called the exception (Fig. 5*B*). The visual target was either the exception or the majority. Under the experimental conditions, the participants’ control over the exception or the majority differed by 20%. Three sets of control level were selected: 10% versus 30%, 50% versus 70%, and 80% versus 100%. These sets corresponded to the no-control category, the boundary of control-category boundaries, and the full-control category, respectively. The control level for the exception was 20% more, or 20% less, than the majority (e.g., there were trials where the exception was under 50% control while the majority was under 70% control, and trials where the exception was under 70% control while the majority was under 50% control). At baseline, there were no stimuli under the control of the mouse (all were under 0% control), but the moving speed corresponded to the mouse’s motion. At baseline, the direction of the stimulus was generated by combining two randomly selected sections of a prerecorded motion (for example, consider two other people controlling the stimuli). The first prerecorded motion controlled 50% of the exception (i.e., 50% of exceptions were controlled by the second prerecorded motion) and 30% of the majority (i.e., 70% of the majority was controlled by the second prerecorded motion). In all four conditions, including at baseline, the combination ratios were selected to ensure that the motions of the exception and the majority shared 80% of the movement direction, and moved in different directions for the remaining 20%. This resulted in a constant 20% visual difference between the exception and the majority, under all four conditions.

In summary, there were two types of visual target: the majority or the exception × [three comparisons (10% vs 30, 50% vs 70%, 80% vs 100%) × two exception control types (more or less than the majority control) + one baseline] = 14 conditions, each condition repeated 15 times, and a total of 210 trials were performed. The trial sequence was randomized for each participant. In addition, participants performed eight practice trials, with two control conditions, each repeated twice. One was the baseline condition, and the other condition controlled the exception and the majority at 40% and 60%, respectively.

#### Data analysis

Trials with reaction time (RT) exceeding ±3 SD from individual average were excluded from analyses (3.8% of all the trials). The difference in RT between the exception (when the target was the exception) and the majority (when the target was in the majority) conditions was used as the index for how much attention was automatically captured by the perceptual difference between the two. A repeated-measures ANOVA (comparison: 10% vs 30%, 50% vs 70%, 80% vs 100%) was used to examine whether visual attention was equally captured by the 20% difference in control in the above three conditions. In case of significant main effect, Bonferroni correction of *p* value was used for *post hoc* comparisons. Furthermore, the three experimental conditions were also compared with individual baseline conditions using paired *t* tests. A Bonferroni-adjusted significance of 0.017 was used for the three comparisons.

### Results

#### Influence of control categories on early attentional allocation

Experiment 1 revealed the typical phenomenon of categorical perception when people detect a change in control. However, it was still unclear whether sensorimotor signals are categorically processed when they have not been transformed into subjective categories of control. Experiment 2 focused on the categorical perception of sensorimotor processing at lower levels, when the judgment of control at higher cognitive levels was unnecessary for the task. The strategy for investigating the effect of categorical perception of control on visual attention allocation was as follows. Theoretically, exceptions automatically capture visual attention; therefore, detection is quicker if the visual target is the exception (Fig. 5*B*, target is the exception). On the other hand, if the visual target belongs to the majority, detection takes longer (Fig. 5*B*, target is one of the majority). The difference in detection time between exception and majority conditions should reflect the perceptual difference between exceptions and majorities (Fig. 5*B*, bottom). Therefore, the difference in RT between the exception (when the target was the exception) and the majority (when the target was in the majority) conditions was used as the index for how much attention was automatically captured by the perceptual difference between the two. This study investigates whether this index is affected by the difference in control between the majority and exception within the same category or at category boundaries.

Figure 5*C* shows the change in the index of attentional capture, according to whether the difference between the majority and the exception was within-category or at the category boundaries. A repeated-measures ANOVA (comparison: 10% vs 30%, 50% vs 70%, 80% vs 100%) revealed a significant main effect of comparison (*F*_(2,38)_ = 12.36, *p *< 0.001, partial η^2^ = 0.39). Bonferroni-adjusted *post hoc* comparisons showed that the effect of target type was significantly greater in the 50% versus 70% condition than the 10% versus 30% and 80% versus 100% conditions (*p *=* *0.010, *p* < 0.001, respectively). The results reveal categorical perception at the sensorimotor level, even when the judgment of control was unnecessary for the task. When the level of control was at the boundary of the control categories, a 20% sensorimotor difference automatically captured greater attention, compared with when the 20% sensorimotor difference was within the category of control.

To determine whether control had a boosting or an inhibitory effect on attention, in addition to the visual difference between the majority and the exception, the performance in each of the three experimental conditions was compared with the performance at baseline. Specifically, when participants had no control over the stimuli at baseline, target type’s (majority vs exception) effect on RT should only be because of the visual differences between target types. On the other hand, in the three experimental conditions where participants had some control over the stimuli, target type’s effect on RT should be a combination of visual and sensorimotor correlation differences. There was no significant difference between baseline and the 50% versus 70% control condition (*t*_(19)_ = 2.37, *p *=* *0.029, Cohen’s *d* = 0.53), but the effects under the other two conditions were significantly smaller than at baseline (for 10% vs 30%, *t*_(19)_ = 6.26, p < 0.001, Cohen’s *d* = 1.40; for 80% vs 100%, *t*_(19)_ = 6.02, *p *< 0.001, Cohen’s *d* = 1.35). The results showed that control had an inhibitory effect on processing the difference between the exception and the majority.

## Discussion

The present study reveals categorical perception of control by showing high discrimination sensitivity between two different levels of control when the comparison is at the boundary of the control categories, rather than when they are within the same control category, controlling for the distance of sensorimotor input. In Experiment 1, participants determined whether their control of a moving dot changed during the trial. Reflecting sensitivity to a 20% change while controlling sensorimotor input, the d′/ΔE was largest when the initial control was at an intermediate level, which corresponded to the boundary of the control categories. Furthermore, Experiment 2 showed that a small difference in sensorimotor input captured more visual attention at the boundary of the control categories than within each control category, even when explicit judgment of control was task irrelevant. In summary, similar to the static features of the self, such as self-voice, name, and face, control, which is a dynamic extension of the self, is also categorically organized as self versus other.

The results of control rating in Experiment 1 showed a typical logistic-like function of subjective judgment of control basing on continuous sensory input (Fig. 4*B*). Such logistic-like function of subjective judgment can be observed in many modalities and perceptions, especially when binary judgment is used. In contrast, some previous studies also reported a linear-like control rating basing on continuous sensorimotor discrepancies (Saito et al., 2015; Wen et al., 2015; Krugwasser et al., 2019). However, the steepness of the function of control judgment was not the focus of the present study. The most important feature of categorical perception is its influence on perceptual sensitivity (i.e., the hypothesis shown in Fig. 1, middle panel). Categorical perception diminishes perceptual sensitivity to within-category difference, while enhancing perceptual sensitivity at the boundary of categories. The mechanism allows people to process complicated sensory input with lower cognitive loads and provides us with proto-symbolic thinking (Goldstone and Hendrickson, 2010). Our results showed that the perceptual processing of sensorimotor input was shaped by the subjective categories of control, supporting our hypothesis that the perception of control is categorical, shown in Figure 1.

A recent study that examined the timeline of how the brain processes sensorimotor signals (i.e., spatial discrepancies) for self-other attribution reported that the bilateral precentral gyri and left inferior parietal lobe (IPL) process sensorimotor discrepancies at an earlier stage, and the right supramarginal gyrus, which is a portion of the IPL, represent the information sensitive to self-other attribution at a later stage (Ohata et al., 2020). In addition to an understanding of the neural basis of sensorimotor processing and cognitive judgment of control, the findings of categorical perception of control further reveal an interplay between cognitive judgment of control and perceptual processing of sensorimotor signals. The information flow is probably not one-way from sensorimotor processing to cognitive judgment.

Experiment 1 revealed that discrimination sensitivity is high between categories but that the sensitivity is low within a category, which was predicted by our main hypothesis that perception of control is categorical. Moreover, Experiment 1 also revealed an interesting phenomenon in within-category discrimination. That is, the initial control affects sensitivity to small changes in control when the changes occurred within a category. If individuals already had a relatively high level of control (within the full-control category), a 20% increase in control (i.e., from 80% to 100%) was ignored, while a 20% decrease in control (i.e., from 100% to 80% control) was significant, despite the fact that the comparisons of sensorimotor input were the same in both cases. This was the case when the no-control category was initially formed, where an increase from 20% to 40% control was detectable, but a decrease from 40% to 20% control was ignored. This phenomenon is consistent with previous studies of categorical perception that examined within-category changes. Once a subjective category has been formed, changes in toward the prototype of the category are less detectable than changes toward the boundaries (Etcoff and Magee, 1992; Acker et al., 1995). Further, a recent study on the sense of control also reported that initial exploration of control greatly influences the detection sensitivity of changes in the subsequent sensing of control (Wen et al., 2020). Moreover, previous studies reported both that lack of control is salient (Blakemore et al., 1998; Timm et al., 2014) and that control captures attention (Salomon et al., 2013; Wen and Haggard, 2018). Here, categorical perception of control presents a framework for understanding these phenomena; suggesting that differences in control potentially cause determinant changes in the control category is significant. When the initially-formed category (i.e., the default category) is full control, a lack of control captures attention. On the other hand, when the initially formed category is no control, gaining control is then significant.

Furthermore, Experiment 2 shows that differences in control at the control-category boundaries attracted early visual attention more than those within each category of control. The results may be explained by the effect of the automatic processing of the sense of control on attentional allocation, even when task irrelevant. Future research should consider this issue. Furthermore, Experiment 2 showed that this effect of categorical perception was mainly because of the inhibition of early attention allocation to differences within the same category (i.e., the comparisons of 10% vs 30% control and 80% vs 100% control). The within-category sensorimotor differences significantly decreased the effects of the target type on RT in the 10% versus 30% and 80% versus 100% control comparisons. However, the overall controllability of the baseline condition (i.e., 0% overall control in the baseline) did not match other conditions (i.e., higher overall control), making it unclear whether the differences between the baseline and other conditions were because of a nonzero difference in control between the exceptions and majority or because of different overall controllability. Follow-up experiments with different levels of overall control to match the three experimental conditions can be used to further examine this issue.

In summary, this study supports the hypothesis of categorical perception of control for both the sense of control at a cognitive level and the implicit processing of sensorimotor input. When individuals interact with the external world, sensorimotor input can vary dramatically. Categorical perception helps our cognitive system allocate resources to the most critical quantitative changes in sensorimotor input. Categorical perception of control also allows us to meaningfully process the relationship between actions and changes in the external world, and these meaningful representations of control, in turn, affect our sensitivity to the consequences of our actions.

## Synthesis

Reviewing Editor: Li Li, New York University Shanghai Decisions are customarily a result of the Reviewing Editor and the peer reviewers coming together and discussing their recommendations until a consensus is reached. When revisions are invited, a fact-based synthesis statement explaining their decision and outlining what is needed to prepare a revision will be listed below. The following reviewer(s) agreed to reveal their identity: NONE.

Both reviewers agreed that the topic is interesting and the research is timely. However, both have concerns that should be addressed before the ms can be considered for publication. Both also agreed that no additional experimental data need to be collected and the required revision can be completed within 2 months. Please see their detailed comments below.

Reviewer 1

The authors of this manuscript tested the effect of categorization on the sense of control. Previous studies have modeled Sense of control using sensorimotor signals. It is still not clear how our brains connect the continuous sensorimotor signals and the cognitive judgement of control. The authors tested if the relationship between the sense of control (i.e., perception) and the sensorimotor (i.e., physical) input is nonlinear because of categorization. They also tested the effect of automatic capture of attention on reaction time in a visual search task. The authors concluded that the categorization in sense of control affects the sensitivity of control even unconsciously.

Major comments about Introduction

The authors talked about how the self is identified or how the scope of self is distinguished in the first paragraph of Introduction. I thought that the authors are interested in the categorization between self and others in actions. However, the authors did not come back to the topic of ‘self’ in the rest of this manuscript. I am wondering if the first paragraph just makes it difficult for readers to have an idea of the question that the authors want to solve.

The paragraph describing the framework was also confusing. Readers naturally look at the leftmost part of Figure 1 (categorization process of perception of control at the physical level) first but the paragraph describing about Figure 1 describes the advantage of categorization first and the underlying mechanisms which make this advantage possible at the perceptual level second. This flow of explanations makes it difficult for readers to follow the flow of this paragraph.

The flow of the last half of this paragraph was also difficult to follow. The authors wrote two questions from line 79 to 85 (they called the second one “hypothesis” but actually they wrote two questions) but I suppose that the authors wanted to describe a hypothesis to answer the question how the continuous sensorimotor signals and the cognitive judgement of control are linked written earlier. I think the connection between their research question and their hypothesis was not clear. They could write here (lines 79-85) that they hypothesize that the categorization at the cognitive level influences the sensitivity of sensorimotor processing at the perceptual level and thus the relationship between the sense of control (i.e., perception) and the sensorimotor (i.e., physical) input is nonlinear.

Major comments about Experiment 1

It was not clear for me if the participants were required to move the mouse (or the dot) freely but I suppose that the task was free moving of the mouse because the authors did not mention about target to follow or reach.

I am wondering if the sense of control is affected by the required precision and/or the level of attention of the tasks. Free moving tasks would not ask high precision.

The authors wrote that the results of Experiment 1 support the hypothesis illustrated in Figure 1. However, it is not clear why it could be expected that the sensitivity to detect the change in control is affected by the direction of the change. Figure 1 (center) only explains that the sensitivity is expected to be low within a category (regardless of the direction of change in action control).

Major comments about Discussion

It was not clear if the results of this study add a leftward arrow from Cognitive level to Perceptual level in Figure 1 or this figure should be kept as it is.

Minor comments

Line 84

Between -> among

Line 316

I suppose they meant “was not significantly different from zero”.

Line 366

were -> where

Line 482 to 484

What this sentence means is not clear.

Reviewer 2

This manuscript explores a very interesting topic and uses a strong choice of experimental methods, to yield relatively convincing results. Below are some comments for improving the clarity of the manuscript:

MAIN COMMENTS

- The baseline in experiment 2 does not entirely make logical sense. The purpose of this baseline seems to be to provide a control so that the sensory aspect of the task (physical difference between the majority and exception dots) can be separated from the control-related aspect of the task. However, the baseline that is used is a 0% control stimulus, where both the majority and exception dots are 0% controllable. This stimulus differs from the experimental conditions in two ways: 1) the difference in controllability between the majority and exception dots is different and 2) the average controllability of the entire display is different. Therefore, it is not clear whether the differences between the baseline and the experimental conditions is due to 1) the experimental condition having a nonzero difference in control between the majority and exception dots, or due to 2) the stimulus having a higher overall controllability than the baseline stimulus.

- One way to address this would be to collect new data in which there are three separate “baseline” conditions, each with a different level of overall control to match the three experimental conditions. For instance, the baseline for the 10% vs 30% condition would be a display where all the circles are 20% controllable. Alternatively, the average performance on each condition could be measured (rather than taking RT difference, simply averaging across all trials). If this average performance is similar across the experimental conditions, this will suggest that overall controllability of the stimulus display does not influence performance.

- It would be helpful to clarify the criteria for a “categorical” representation. If the representation deviates from linearity at all, does that make it categorical? Or, does it need to be perfectly separated into two discrete categories? If the representation were perfectly categorical , then subjects in the control rating task should never report “partial control” and the PSE for reporting no-control and full-control should be closer together (Figure 4D, the yellow area would be smaller). The data shown here appear to be a moderate case, somewhere between perfectly categorical and a continuous representation. These criteria and their relation to the data should be clarified.

- Some of the language is over-stated and over-interpreted. For instance, on line 540 the authors state that the study “proves” the validity of categorical perception; to be more accurate they should use “support” or “suggest” instead of prove. Similarly, in the abstract (line 13), the text reads “cognitive systems are designed to be highly sensitive...” but the results don’t say anything about what the system was designed for, this could read simply “cognitive systems are highly sensitive...”

- Methods section would benefit from re-organizing and streamlining, as follows:

- It would be helpful to have a dedicated section describing the generation of all the motion stimuli, particularly the use of the pre-recorded motion stimuli. Give details like what task is the pre-recorded motion from, and what is the rationale for using this instead of random angular values (some of these details are given later, but they should all be consolidated early in the methods section).

- It would help to use sub-headings for the stimuli and tasks (“Motion stimuli", “Control discrimination task", “Control rating task", etc.)

- The portion of the text directly after the Experiment 1 heading (starting line 107) seems like it should be labeled under Methods, the Methods heading should be moved up to directly under the Experiment 1 heading.

- There is a lot of repetitive text in the methods for Experiment 2 (pages 25-27), for instance with regard to the 20% difference in sensorimotor input and the description of the baseline conditions. This section could be streamlined by removing some repeated statements.

MINOR COMMENTS

- In the results for Experiment 1, it would be better to present Figure 5 before Figure 4C, since Figure 5 introduces the delta E measure which is used to correct d’ (plotted in 4C). One way to accomplish this would be to combine Figure 5 and Figure 4C into a new Figure 5. Figure 4D is referenced in the text before Figure 4C, so it should be shown before Figure 4C.

- For Experiment 1, the results of a repeated measures ANOVA are reported twice - once starting at line 264, and once at line 284. My interpretation is that the first test is performed on the raw d’ values while the second is performed on the values of d’/E. If this is correct, the first test may not be necessary - simply reporting the results of the second test would be sufficient. In any case, this should be clarified.

- In Figure 4C and Figure 6C, it would be helpful to add asterisks or some other symbol to indicate the significance of the post-hoc pairwise tests that were done. This would aid in interpreting these figures with the related text.

- In Figure 4C, is there a significant difference between the increase and decrease conditions, at any control level? From the figure, it looks like 60% vs. 80% might be larger for decrease than increase. If those tests are significant, it would add to the argument that detection is better toward the category boundary.

- The rationale for experiment 2 should be more thoroughly laid out in the introduction. Specifically, it would help to add citations to relevant work on attentional capture, to justify the use of attentional capture as an index of perceptual dissimilarity. Some of this discussion is done in the results and discussion section, but should be introduced earlier.

- Data for the baseline condition in Experiment 2 should be shown.

- The use of the phrase “sensorimotor correlations” on pages 31-32 is a bit confusing since this term hasn’t been used earlier in the text. It would be better to stick to the “control” terminology to be consistent with the earlier text.

- Since the same subjects were used for Experiment 1 and Experiment 2, does the amount of categorical perception in experiment 1 predict the result of experiment 2 for individual subjects?

- Some experimental details should be clarified, as follows:

- In Experiment 1, what precisely are subjects instructed to do - move the mouse around randomly? How much and how fast do typical subjects move the mouse? Are there differences in mouse movements across subjects that relate to their behavior?

- What is the pixel range of the screen where dots can be drawn?

- How many trials are there in the control rating task?

- In the Results section, some specific details such as the Bonferroni correction procedure (line 297) and the version of Matlab that was used (line 253) are included. These very specific details should be moved to the Methods section.

- On line 316, should read “80% to 100% control was not significantly different”?
